# From fighting depression to conquering tumors: a novel tricyclic thiazepine compound as a tubulin polymerization inhibitor

**DOI:** 10.1038/cddis.2016.53

**Published:** 2016-03-17

**Authors:** Y Mu, Y Liu, J Xiang, Q Zhang, S Zhai, D P Russo, H Zhu, X Bai, B Yan

**Affiliations:** 1 School of Chemistry and Chemical Engineering, Shandong University, Jinan, Shandong, China; 2 The Center for Combinatorial Chemistry and Drug Discovery of Jilin University, The School of Pharmaceutical Sciences and The College of Chemistry, Jilin University, Changchun, Jilin, China; 3 The Rutgers Center for Computational and Integrative Biology, Camden, NJ, USA; 4 Department of Chemistry, Rutgers University, Camden, NJ, USA

## Abstract

A novel tricyclic thiazepine derivative, 6-(p-tolyl)benzo[f] pyrido[2,3-b][1,4] thiazepine 11,11-dioxide (TBPT), exhibits potent inhibitory effects in two non-small-cell lung cancer cell lines, H460 and its drug-resistant variant, H460_TaxR_, while exhibiting much less toxic effects on normal human fibroblasts. After five injections of TBPT at a dose of 60 mg/kg, it inhibits H460_TaxR_ tumor growth in xenografted mouse models by 66.7% without causing observable toxicity to normal tissues. Based on gene perturbation data and a series of investigations, we reveal that TBPT is not a P-glycoprotein substrate and it inhibits microtubule formation by targeting tubulin, thereby causing cell cycle arrest at the G2/M stage and eventually inducing apoptosis. This redeployment of anti-depressant compound scaffold for anticancer applications provides a promising future for conquering drug-resistant tumors with fewer side effects.

Drug resistance and nonselective toxicity have been two major clinical obstacles for cancer chemotherapy.^1, 2, 3, 4, 5^ Drug resistance is responsible for treatment failure in >90% of patients with metastatic cancers.^6^ A major cause of drug resistance is the overexpression of a 170-kDa transmembrane protein, P-glycoprotein (P-gp), on the cancer cell surface in response to drug treatment. P-gp acts as an ATP-dependent drug efflux pump.^7, 8, 9^ Many drugs, including paclitaxel (PTX), taxanes, doxorubicin and vinca alkaloids are substrates of P-gp and are readily pumped out of cells, thereby leading to reduced drug accumulation inside cells.^1, 10^ Furthermore, anticancer drugs often elicit serious nonselective toxicity to normal organs. Patients who have undergone chemotherapy often suffer from treatment-related morbidity or mortality, such as leukopenia, hepatic toxicity and even death.^11, 12, 13, 14^ Therefore, potent and selective agents against multidrug-resistant cancers are urgently needed.

Tricyclic azepine derivatives, such as tianeptine, clomipramine and quetiapine, have been used as anti-psychotic drugs. Other reports have demonstrated that tricyclic azepine derivatives such as nevirapine work as anti-human immunodeficiency virus agents.^15, 16^ A recent report has also demonstrated that tricyclic anti-depressants such as clomipramine exhibit anticancer activity.^17^ In this work, we report the good activity and superb selectivity of a tricyclic thiazepine compound, 6-(p-tolyl)benzo[f] pyrido[2,3-b][1,4] thiazepine 11,11-dioxide (TBPT) against drug-resistant non-small-cell lung cancer (NSCLC). TBPT inhibited the growth of both drug-sensitive and drug-resistant human lung cancer cells *in vitro* and drug-resistant tumors *in vivo* without obvious side effects. Further examination indicated that TBPT evaded the P-gp-mediated drug efflux and acted as a novel microtubule depolymerizing agent, thereby inducing cancer cell G2/M phase arrest and apoptosis.

## Results

### Selective cytotoxicity against H460_TaxR_ and H460 cells

**Figure 1:**
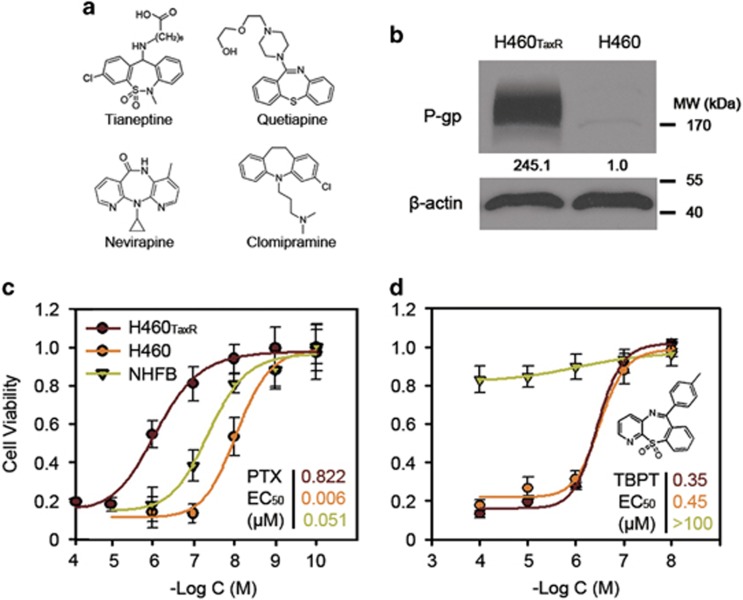
(**a**) The representative structures of bioactive tricyclic derivatives reported previously. (**b**) The P-gp expression in H460_TaxR_ and H460 cells. (**c** and **d**) The dose-dependent cytotoxicity of PTX or TBPT. The cytotoxicity of PTX (**c**) or TBPT (**d**) were analyzed in NSCLC cell lines H460_TaxR_, H460 and normal fibroblasts cell line NHFB (*N*=3). The EC_50_ of compounds were shown in the corresponding colors of cell lines

Tricyclic azepine derivatives were bioactive for several diseases, especially depression (Figure 1a).^15, 16, 17^ In an effort to redeploy anti-depressant compound scaffold for anticancer applications, we synthesized a series of 27 novel tricyclic thiazepine derivatives^18^ and screened these compounds against the NSCLC cell line H460 and its drug-resistant variant H460_TaxR_ (manuscript submitted).^19, 20, 21^ The drug-resistant H460_TaxR_ cell line was obtained by treating H460 cells initially with 5 nM PTX and increased doses up to 100 nM, leading to a remarkable overexpression of P-gp (245-folds, Figure 1b) among other possible alterations. Compared with H460, H460_TaxR_ is highly resistant to many clinical drugs, including PTX, vincristine and doxorubicin, because of the drug pump P-gp (Figure 1c, Supplementary Table S1). In contrast, 1 of the 27 derivatives, TBPT, exhibited the most potent cancer cell inhibitory activity with a half maximal effective concentration (EC_50_) <0.5 *μ*M against both H460_TaxR_ and H460 cells, whereas exhibiting minimal toxicity toward normal human fibroblasts (NHFBs; EC_50_>100 *μ*M; Figure 1d). These results showed the cytoselective anticancer activity of this compound.

### Drug-like properties of hits

As *in vivo* investigations are generally time consuming and expensive, it is advantageous to eliminate those compounds that are not suitable for animal experiments. One of the necessary steps is to screen the initial hits using assays testing their possible behaviors *in vivo*. To evaluate the drug-like properties of TBPT, a panel of *in vitro* assays was preformed (Table 1). The parallel artificial membrane permeability assay (PAMPA) was used to evaluate the cell permeability of the compounds. Compounds with a Pe >200 cm/s are classified as highly permeable. TBPT exhibited excellent potential cell permeability. The Caco-2 assay was used to predict the oral absorption values of the compounds *in vivo*. Compounds with a Caco-2 efflux ratio >2 may be subject to active efflux and should not be developed further. TBPT had an efflux ratio of 0.4–0.8. The half-life (T_1/2_) in human liver microsomes is used to predict the metabolism properties of compounds *in vivo*. A very small T_1/2_ suggests quick metabolism in the liver, and compounds with small values should be avoided. A very long T_1/2_ (>24 h) increases the risk of cumulative toxicity and is not desirable. TBPT exhibited suitable T_1/2_ values in liver microsomes. T_1/2_ in plasma indicates the stability of the drug in human blood; the high T_1/2_ values of these compounds indicated better stability *in vivo*. Plasma protein binding influences the distribution of drugs into body tissues, and compounds with high plasma protein binding (>99%) are limited in terms of the amount of free compound that are available to act on the targeted tissue. As indicated in Table 1, TBPT exhibited overall suitable drug-like properties.

### Inhibition of the growth of NSCLC xenografts in mice without appreciable toxicity to normal tissues

**Figure 2:**
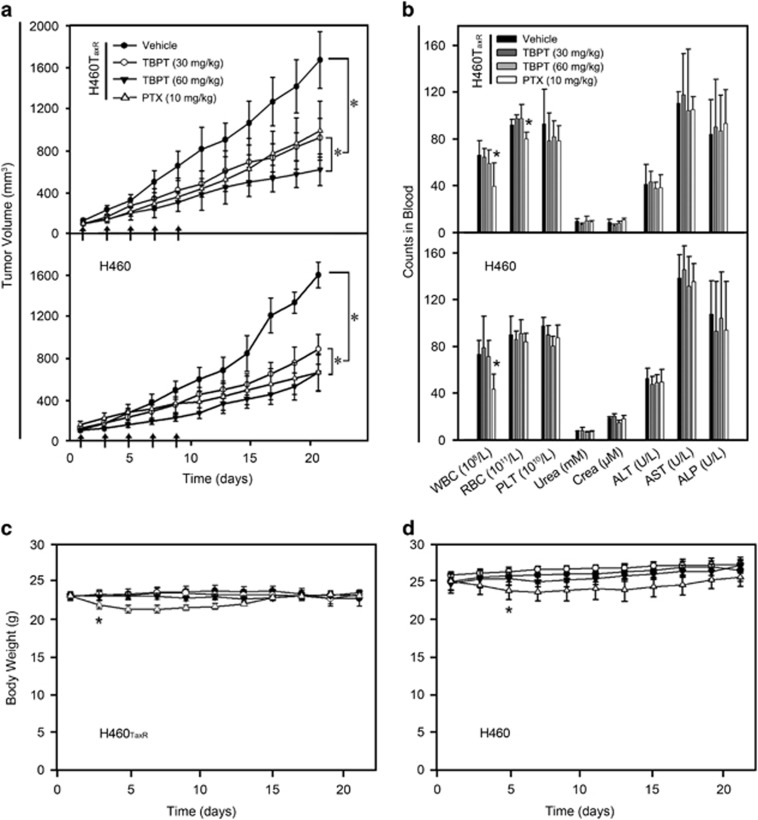
TGI by TBPT in H460_TaxR_ and H460 xenograft mouse models. Five injections of vehicle and TBPT (30 or 60 mg/kg) were administered intravenously at days 1, 3, 5, 7 and 9 (arrows). Four injections of PTX (10 mg/kg) were administered intravenously at days 1, 3, 5 and 7. (**a**) The tumor volume was measured every other day. When the tumor volume in the solvent-treated group exceeded 1500 mm^3^, the experiment was terminated. (**b**) Blood biochemical and hematological analyses. (**c**) The body weights of the mice were monitored every other day during the experiments. *P<0.05, Student's *t-*test with day 0, *N*=5

**Figure 3:**
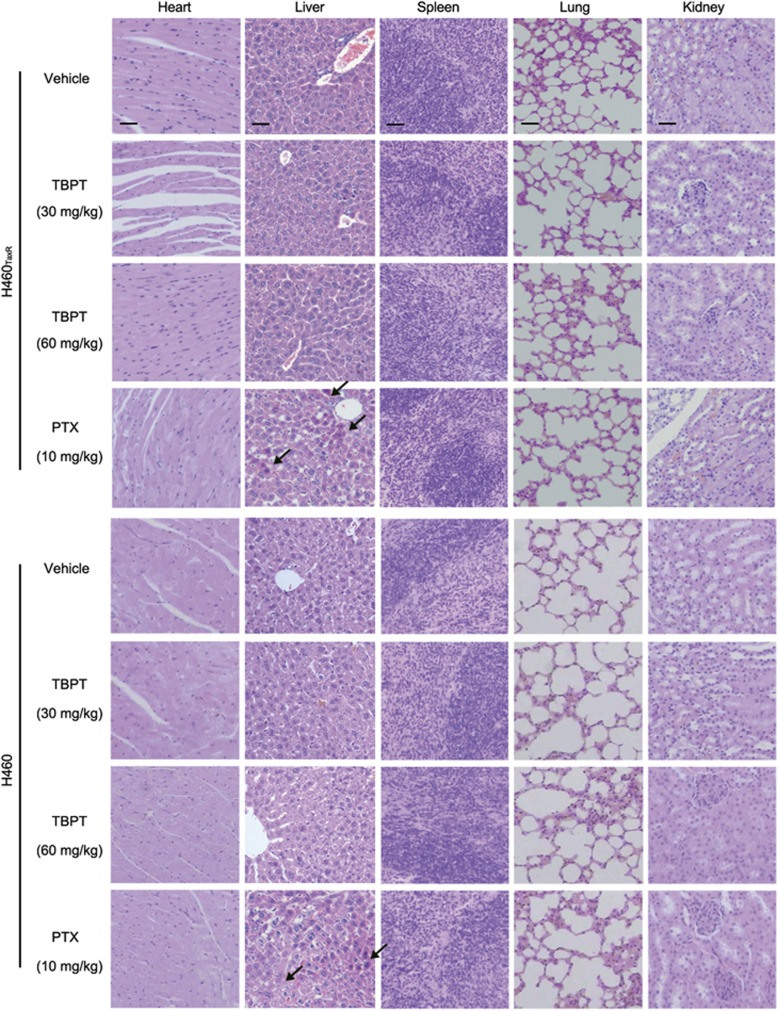
Histopathological examination of the major organs from TBPT or PTX-treated mouse tumor at the end of the experiments. The cross-sections of organs were stained with hematoxylin–eosin. No pathological changes were observed in any group of TBPT-treated mice. Hepatocyte necrosis was observed in some areas in the livers of PTX-treated mice (arrows). The scale bars represent 50 *μ*m and are uniform in each column

Based on cell inhibition data, TBPT was then investigated for its tumor inhibitory property in mouse models. Five doses of TBPT (60 or 30 mg/kg) or four doses of PTX (10 mg/kg) were administered every other day to H460_TaxR_ or H460 xenografted mice. Owing to drug resistance in H460_TaxR_ tumors, PTX exhibited less tumor inhibitory effect in H460_TaxR_ model (tumor growth inhibition, TGI=42.0%) compared with that in H460 model (TGI=66.2%). Application of PTX, however, caused significant decreases of white blood cells, red blood cells and body weights of the mice, which indicated a systematic toxicity. Hepatocyte necrosis was also observed in the livers of mice treated with PTX (Figures 2 and 3). Five injections of TBPT (60 mg/kg) caused similar TGI in both H460_TaxR_ and H460 tumors (TGI values of 66.7% and 62.9%, respectively), whereas TBPT did not exhibit evident toxicity to normal tissues based on toxicity indicators, such as blood biochemical and hematological analyses, body weight change (Figure 2), relative organ weights (Supplementary Figure S1) and histopathological examination of the major organs (Figure 3). Taken together, these results show that TBPT-induced potent tumor inhibitory effects in H460 xenografts and drug-resistant H460_TaxR_ xenografts without obvious side effects. The drugs of clinical chemotherapy, such as PTX, carboplatin and vincristine, generally lead to systematic toxicity, including appetite loss, sensory neuropathy, hematologic toxicity, hepatic/renal toxicity and even death.^11, 12, 13, 14, 22, 23, 24^ The superb tumor selectivity of TBPT *in vitro* and *in vivo* indicated that it might cause less toxicity when applied in clinical.

### TBPT is not a P-gp substrate

**Figure 4:**
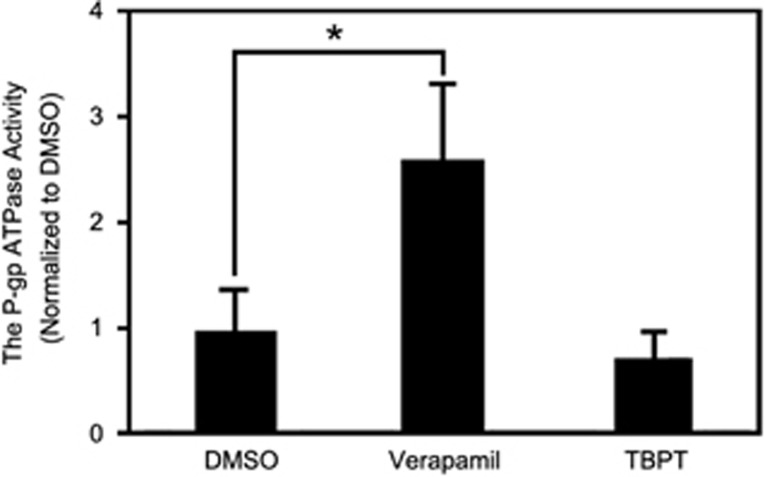
TBPT is not a P-gp substrate. The P-gp ATPase activity was determined in human cell membrane P-gp fractions using a luminescence-based assay. Verapamil, a known P-gp substrate, was used as a positive control and increased P-gp ATPase activity significantly. *N*=3

To determine how TBPT overcomes the drug resistance of H460_TaxR_ tumors, we investigated whether TBPT was a substrate of P-gp using a P-gp ATPase activity assay. Substrates of P-gp, such as verapamil, typically stimulate P-gp ATPase activity.^25, 26^ Compared with the negative control dimethyl sulfoxide (DMSO), verapamil increased the activity of P-gp ATPase by approximately threefold, whereas TBPT did not induce P-gp ATPase activity (Figure 4). These results showed that TBPT was not a substrate of P-gp and was not subjected to being transported outside. This finding explains why TBPT maintains potent inhibitory activity in drug-resistant H460_TaxR_ cells and tumors. Substrates of P-gp are structurally diverse, including many anticancer drugs,^8^ HIV protease inhibitors^27^ and cardiovascular drugs.^28^ There is still no clear understanding on common structure features of P-gp substrate. However, from experimental screening and testing, compounds like thiazolidinone and TBPT are discovered to escape P-gp transport.^29, 30, 31, 32^ Such compounds with anticancer activity may help overcome drug resistance in chemotherapy of cancers.

### TBPT-induced perturbation of gene expression in H460_TaxR_ cells

Although TBPT is not a substrate of P-gp and is allowed to maintain a high concentration inside drug-resistant cancer cells, its cellular target(s) remain unknown. To explore the possible targets of TBPT, we determined the global gene expression profile of H460_TaxR_ cells after TBPT treatment using an Agilent whole-human genome oligo microarray platform.^20^ The microarray analysis revealed that 534 genes were suppressed and 410 genes were upregulated (≥2-fold) in H460_TaxR_ cells following TBPT treatment (2 *μ*M, 4 h). The gene ontology analysis indicated that genes involved in regulating microtubule cytoskeleton and nucleus functions were altered by TBPT (Supplementary Table S2). The corresponding affected biological processes included microtubule cytoskeleton organization, microtubule-based movement, spindle localization, mitotic cell cycle process, chromosome segregation and DNA/RNA metabolic processes (Supplementary Table S2). Moreover, because TBPT shares a similar scaffold with tricyclic anti-depressant drugs, it also upregulated neurological system process genes, such as *GNAT1*, *NPAS3*, *GSX2* and *SCN8A* (Supplementary Table S2). These results suggested that TBPT might influence the microtubule organization process.

### TBPT inhibited tubulin polymerization in a cell-free system and in live cells

**Figure 5:**
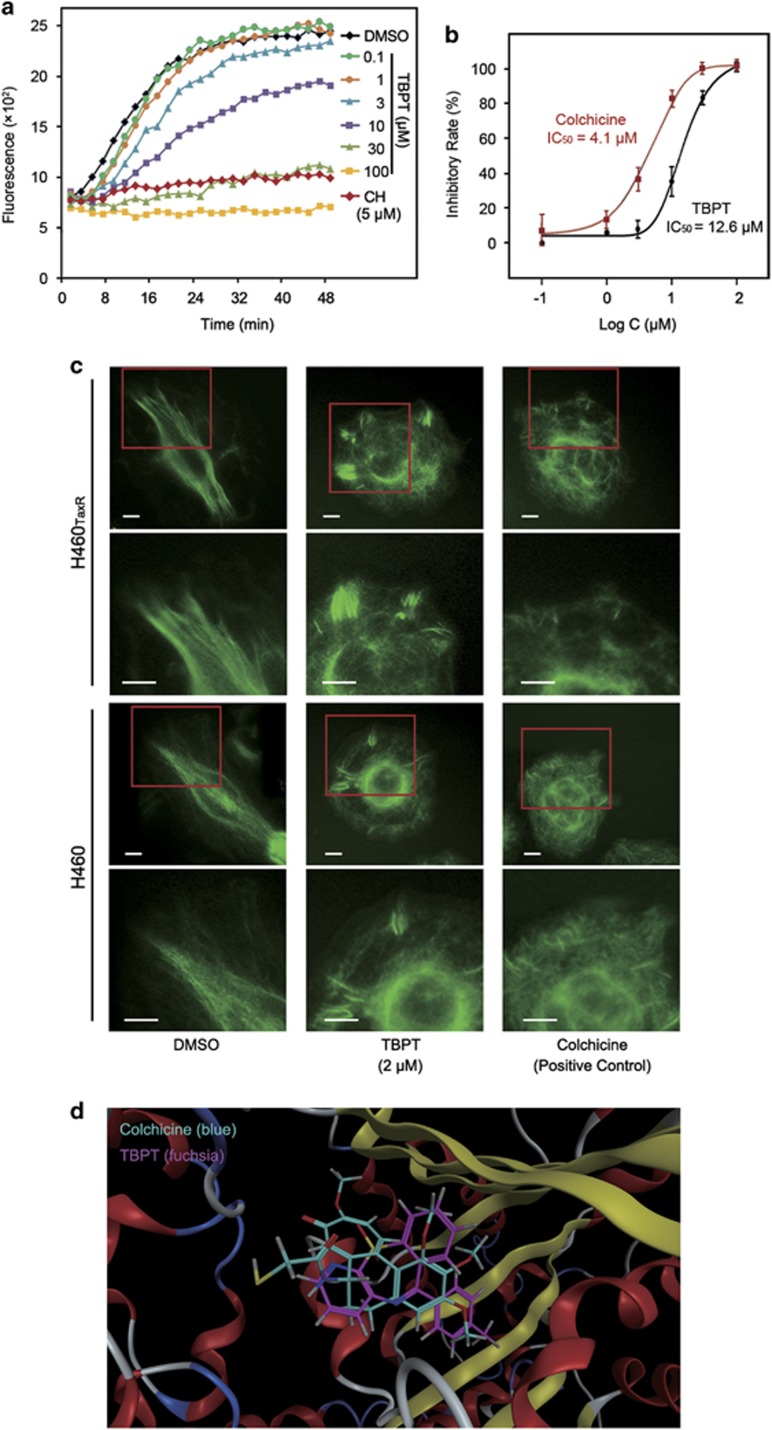
TBPT inhibited tubulin polymerization and disrupted microtubule formation. (**a**) The rate of the tubulin polymerization process was monitored via fluorescence in the presence of TBPT or colchicine (CH) at the indicated concentration in a cell-free system. The fluorescence was monitored every 2 min for 50 min. (**b**) Dose-dependent tubulin polymerization was observed. *N*=2. (**c**) After the cells were treated with DMSO, TBPT or CH for 24 h, their cellular microtubules were visualized via green fluorescence using a fluorescent secondary antibody binding specifically to anti-*α*-tubulin antibody. The scale bars represent 10 *μ*m. (**d**) The molecular docking study of TBPT to the CH-binding site of tubulin

To confirm the DNA array analysis findings, we first assessed the effect of TBPT on tubulin polymerization in a cell-free system^33^ using the well-known microtubule depolymerizing agent colchicine and stabilizing agent PTX as positive controls and DMSO as negative control (Figure 5a, Supplementary Figure S2). The results showed that alpha- and beta-tubulin subunits dimerized and self-assembled to form microtubules in a time-dependent manner after DMSO treatment, as indicated by a steady increase in fluorescence (Figure 5a). Colchicine caused strong inhibition of microtubule formation, with a half maximal inhibitory concentration (IC_50_) value of 4.1 *μ*M. TBPT also inhibited the formation of microtubules, with an IC_50_ value of 12.6 *μ*M (Figure 5b).

As inhibition of tubulin polymerization may cause microtubule disruptions, we next evaluated microtubule organization in live cancer cells. As shown in Figure 5c, fluorescence-labeled microtubules normally display a shape of straight lines in both NSCLC cell lines treated with DMSO (left column). After treatment with TBPT, microtubule damage, such as disordered microtubule fragments, were detected in both drug-sensitive and drug-resistant NSCLC cells (middle column). This damage was similar to that caused by the tubulin inhibitor colchicine (right column).

To better understand how TBPT interacts with tubulin, the molecular docking of TBPT to the colchicine-binding site of the tubulin crystal structure (1SA0) was performed using the triangle matcher placement algorithm. TBPT exhibited good matching with the colchicine-binding site, with a docking S score of –5.69, which was extremely close to that of colchicine (−6.02). As shown in Figure 5d, TBPT (fuchsia) overlapped well with colchicine (blue) in the docking model. It was indicated that TBPT might bind with tubulin at or near the colchicine-binding site.

These results and results from the previous section demonstrated that TBPT functions as a inhibitor for tubulin polymerization. Microtubule-targeting agents work as anti-mitotic agents, causing cell cycle arrest and apoptosis.^34^ However, most known microtuble-targeting agents, such as PTX,^35^ vinca alkaloids^36^ and colchicine,^37^ exhibit intense dose-limiting toxicity with narrow therapeutic window. Microtubule-targeting agent TBPT, with a much lower toxicity, is a promising starting point for developing of a new generation of anticancer agents.

### TBPT-induced G2/M arrest and apoptosis in NSCLC cells

**Figure 6:**
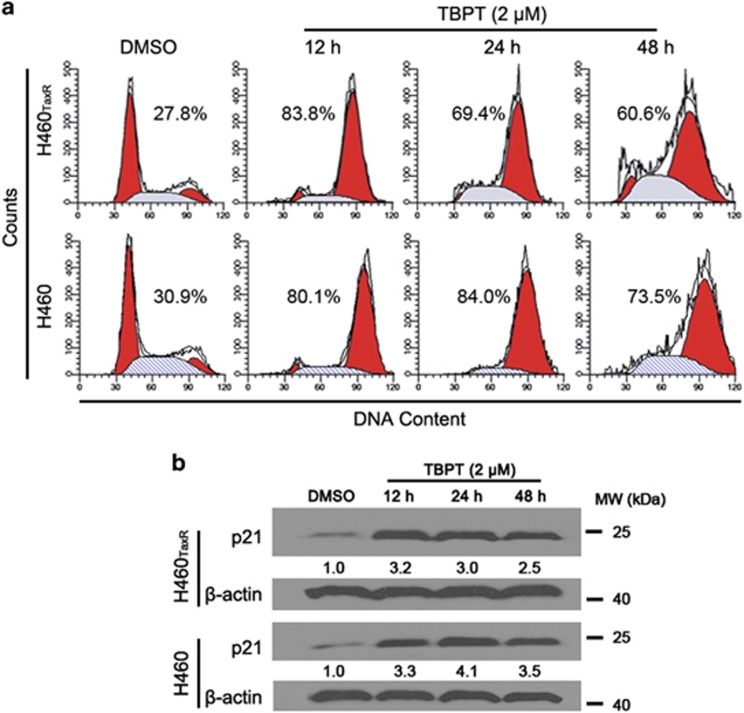
Analyses of the cell cycle and p21 expression in H460_TaxR_ and H460 cells after TBPT treatment. NSCLC cells were treated with DMSO or TBPT (2 *μ*M) for the indicated times. (**a**) Cells were stained with PI and analyzed via flow cytometry. The two red peaks indicate the cells in the G1 and G2/M phases, respectively. The middle gray area indicates the cells in the S phase of the cell cycle. The ratio of cells in the G2/M phase is also shown. (**b**) Western blot analysis of p21 expression in NSCLC cells

Microtubule-targeting agents suppress microtubule dynamics, resulting in mitotic arrest and apoptosis.^38^ As shown in Figure 6a, strong G2/M arrest (over 80% cells) was detected in both H460_TaxR_ and H460 cell lines after 12 h of TBPT treatment. Consistent with this finding, p21 (waf1/cip1), a well-known cell cycle progression inhibitor, was significantly upregulated in TBPT-treated cells (Figure 6b).

**Figure 7:**
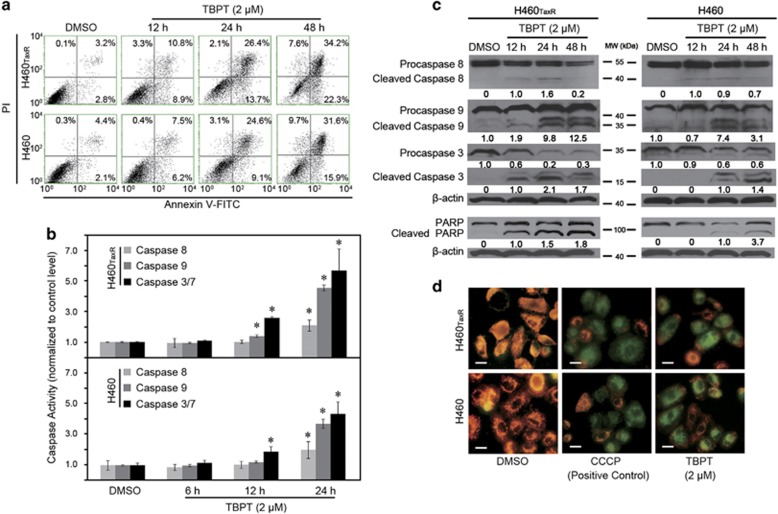
TBPT induces time-dependent apoptosis in both H460_TaxR_ and H460 cells. (**a**) The cells were treated with DMSO or TBPT (2 *μ*M), stained with PI/Annexin V-FITC, and analyzed using flow cytometry. (**b**) The activities of caspases 8, 9 and 3/7 were examined using a fluorescence-based assay. *Student's *t*-test with the DMSO group, *P*<0.05, *N*=3. (**c**) Western blot analysis of caspases 8, 9, 3 and PARP after TBPT treatment in NSCLC cells. (**d**) The mitochondrial membrane potential was determined using JC-1 dye, and a well-known mitochondrial uncoupler, CCCP, was used as a positive control. Scale bar, 20 *μ*m. Healthy cells maintain a high mitochondrial membrane potential and emit red fluorescence, whereas apoptotic cells have a low mitochondrial membrane potential and emit green fluorescence. The mitochondrial membrane potential decreased significantly in NSCLC cells treated with TBPT

Moreover, approximately 50% of the NSCLC cells were in an apoptotic/necrotic state after a 48-h treatment with TBPT (2 *μ*M; Figure 7a). To further elucidate TBPT-mediated apoptosis pathways, we examined the activities of caspases 8, 9 and 3/7 after TBPT treatment. As shown in Figures 7b and c, TBPT treatment activated caspases 8, 9 and 3/7 in a time-dependent manner in both H460_TaxR_ and H460 cell lines. This result indicates that the TBPT-induced cell apoptosis was the result of activating both extrinsic and intrinsic apoptosis pathways. To validate the occurrence of apoptosis, we also observed the cleavage of PARP and a decrease in the mitochondrial membrane potential after TBPT treatment (Figures 7c and d). In short, TBPT selectively induced G2/M cell cycle arrest and apoptosis in both H460_TaxR_ and H460 cells, which is consistent with the anticancer mechanisms of other reported microtubule-targeting agents, including nocodazole and PTX.^39, 40, 41, 42, 43, 44, 45^ In contrast, TBPT-induced very little cell cycle arrest or apoptosis in NHFB cells and mice hippocampus cells (Supplementary Figures S3 and S4), which is consistent with the observed low cytotoxicity to NHFB cells. Beside TBPT, there are other less toxic microtubule-targeting agents reported with unknown mechanism.^32, 46^

### The likely mechanism of the low toxicity of TBPT to normal cells and organs

Tubulin molecules exist in both cancer and normal cells. However, TBPT selectively inhibits the growth of cancer cells or tumors. Studies have shown that a rapid rate of cell division and a higher turnover of active tubulin dynamics in cancer cells make them acutely sensitive to anti-tubulin drugs.^40, 47^ The moderate inhibitory ability of TBPT against tubulin polymerization (IC_50_=12.6 *μ*M) may maintain cancer inhibition because of its higher microtubule dynamics and exhibit less toxicity to normal cells because of their low microtubule dynamics.^21^ In addition, other targets of TBPT that account for its selective anticancer activity may also exist. For example, TBPT significantly suppressed a series of genes involved in DNA double-strand break repair, including *LIG4*, *CENPF*, *DNAJC2*, *RECQL*, *SMC6* and *BRCA2* (≥ 2-fold; Supplementary Table S2), which are also considered vital targets for treating cancer cells without affecting normal cells.^48, 49^

**Figure 8:**
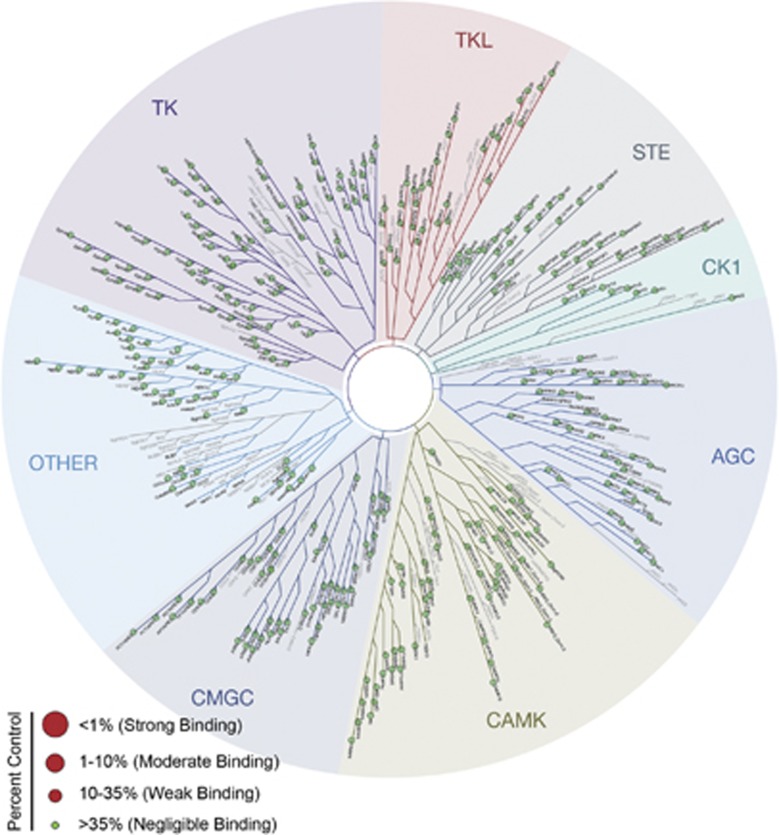
The ability of TBPT to bind and inhibit 442 kinases in the human kinome. TBPT was screened twice at 5 and 10 *μ*M using a kinome scanning assay. The 35% of the binding percentage of DMSO was used as the criterion of negligible binding, as suggested by the manufacturer and previous reports.^60, 61, 62^ If any kinase binds TBPT (%Ctrl<35%), then a large red dot is shown in the kinome tree. The binding and inhibitory percentages for TBPT for all 442 human kinases (covering 80% of the human kinome) were greater than the criterion (35%). The kinase groups are abbreviated as follows: TK, tyrosine kinase; TKL, tyrosine kinase-like; STE, homologs of the yeast sterile 7, 11 and 20 kinases; CK1, casein kinase 1; AGC, the protein kinase A-, G- and C-containing families; CAMK, calcium/calmodulin-dependent protein kinase; CMGC, cyclin-dependent kinase-, mitogen-activated protein kinase-, glycogen synthase kinase 3-, and dual-specificity protein kinase-containing families; OTHER, all other protein kinases that do not belong to specific groups. The detailed results are presented in Supplementary Table S3

In addition, off-target effects of drugs, which are caused by interactions between drugs and unintended targets or signaling pathways, can also disrupt normal functions of cells and cause nonselective drug toxicity.^50, 51, 52, 53^ In eukaryote cells, almost all signaling pathways are mediated by protein kinases, which constitute the largest functional protein family.^54^ Unintended inhibition of protein kinases may help the anticancer activity of TBPT but can also lead to serious nonselective drug toxicities.^55, 56, 57^ To further evaluate the off-target effects of TBPT, we next assessed the ability of TBPT to inhibit a panel of 442 human kinases (covering 80% of the human protein kinome) at 5 and 10 *μ*M using a kinome scanning platform.^58, 59^ TBPT exhibited negligible binding affinity to or inhibitory activity against the 442 human kinases (Figure 8, Supplementary Table S3).^60, 61, 62^ This result further accounted for the lack of off-target effect of TBPT. Therefore, TBPT is a very selective tubulin polymerization inhibitor.

## Discussion

### P-gp-independent anticancer activity of TBPT

In this study, we reported that TBPT exhibited potent anticancer activity in drug-resistant H460_TaxR_ cell line and mice tumor. We revealed that TBPT is not a substrate of P-gp, a major origin of multidrug resistance. This provides the basis for TBPT to enter cancer cells and provides a key explanation for its P-gp-independent anticancer activity. Indeed, there are many other mechanisms of cancer cells to induce drug resistance besides P-gp.^1, 63^ TBPT can overcome the P-gp-mediated drug resistance. This does not mean that TBPT is bioactive for all cancer cells with other drug-resistant mechanisms. However, TBPT has removed the major barrier (P-gp) to interact with cancer cells, which is an advantage compared with other drugs.

### Low toxicity of TBPT *in vitro* and *in vivo*

TBPT exhibited less toxicity to NHFBs compared with cancer cells. Furthermore, no obvious toxicity was found in TBPT-treated mice compared with PTX-treated mice. PTX caused significant decreases of white blood cells, red blood cells and body weights of the mice, which indicated a systematic toxicity. And considering that serious toxicities were detected during PTX treatment, we decreased the drug doses to four injections. TBPT shares a similar scaffold with tricyclic anti-depressant drugs. In addition, it also affected several neurological system process genes, such as GNAT1, NPAS3, GSX2 and SCN8A. But no obvious abnormal behaviors of mice were observed during TBPT treatment. Also, TBPT did not show toxic effects in mice hippocampus cells, based on cell cycle and apoptosis analysis, which indicated that TBPT might have little neurotoxicity when applying in human.

We found that TBPT interacted with tubulin dynamics, which is ubiquitous throughout all animal cells, but TBPT did not cause obvious toxicity. We speculate that TBPT may benefit from its moderate tubulin inhibitory effect, which may not be enough to inhibit normal cells with lower tubulin dynamics (less target concentration) but is lethal to cancer cells with much more active tubulin dynamics (more target concentration). TBPT also benefits from its much less off-target effects according to the kinome assay result. In addition, there may be other critical targets besides tubulin, for example, TBPT significantly suppressed a series of genes involved in DNA double-strand break repair, which is important to selective drug toxicity.^48, 49^

Drugs all have adverse effects, especially for anticancer drugs. We evaluated the general toxicity of TBPT to human fibroblasts and neuron-derived cells, mice body weight, blood analyses and major organs. Further adverse effects should be properly assessed in more details if taking this compound to clinic. However, based on our experimental data, we may conclude that TBPT is much safer than PTX to achieve the comparable anticancer effect.

In conclusion, we redeployed the anti-depressant tricyclic thiazepine scaffold for an anticancer application. Compound TBPT was discovered from a library of tricyclic thiazepine derivatives through a cytoselective toxicity assay. TBPT inhibited the growth of both drug-sensitive (H460) and drug-resistant (H460_TaxR_) NSCLC cells with an EC_50_<0.5 *μ*M, whereas exhibiting less toxicity to NHFBs (EC_50_>100 *μ*M). It inhibited the growth of both H460_TaxR_ and H460 tumors in xenografted mouse models by >60% without appreciable toxicity to normal tissues. We revealed that TBPT evaded P-gp-mediated drug efflux and inhibited tubulin polymerization, leading to microtubule damage, cell cycle arrest in the G2/M phase and cell apoptosis. In conclusion, we discovered a novel tricyclic thiazepine derivative as a tubulin polymerization inhibitor exhibiting potent anticancer activity against drug-resistant NSCLC *in vitro* and *in vivo*. Our finding indicates a bright future of drug redeployment efforts and provides an effective therapeutic solution for drug-resistant tumors.

## Materials and Methods

### Reagents and cell culture

All biological reagents and solvents were obtained from commercial suppliers and used as recommended. The human NSCLC cell line H460 and P-gp-overexpressing drug-resistant cell line H460_TaxR_ were cultured in RPMI-1640 medium supplemented with 10% fetal bovine serum, 100 units/ml penicillin and 100 mg/ml streptomycin. The NHFB cell line NHFB was cultured in DMEM with the same supplements. All cells were maintained in a 37 °C incubator with 95% humidity and 5% CO_2_.

### Cell viability assay

The cell viability was determined using the sulforhodamine B assay.^64^ In brief, cells (4000 cells in 100 *μ*l medium) were seeded in 96-well culture plates and incubated for 24 h. The cells were treated with the compounds or with an equal volume (0.1%) of DMSO for 72 h. The cell viability was calculated using the following formula: cell viability=*A*_*treatment*_*/A*_*control*_. The EC_50_ values were determined using the Sigma Plot 10.0 software package (Systat Software Inc., San Jose, CA, USA).

### *In vitro* drug properties assays

The PAMPA and Caco-2 cell bi-directional transport assay of the derivatives were conducted as described in our previous report.^65, 66^ The human liver microsome, plasma stability and plasma protein-binding assays were conducted following modified protocols reported by Cyprotex (Macclesfield, UK).^67, 68, 69, 70^

### *In vivo* experiments

NSCLC xenograft tumor models were constructed as reported previously.^20, 21^ Four-week-old female athymic nude mice were purchased from Vital River Laboratory Animal Technology Co., Ltd (Beijing, China) and housed under pathogen-free conditions. This study was performed following the recommendations from the Guide for the Care and Use of Laboratory Animals by the National Institutes of Health. The protocol was approved by the Committee on the Ethics of Animal Experimentation of Shandong University. H460 and H460_TaxR_ cells (5 × 10^6^ cells in 100 *μ*l PBS) were injected subcutaneously into the dorsal flank of each mouse. When the tumors reached volumes of 100 mm^3^, the mice were divided randomly into four groups. The mice were treated every other day five times with intravenous injections of (1) vehicle (the solvent of TBPT: 60% PBS, 8% Cremophor EL, 8% EtOH, 12% poly propylene glycol and 12% PEG-400), (2) TBPT (30 mg/kg) or (3) TBPT (60 mg/kg). Another group was treated with PTX (10 mg/kg) every other day four times as the positive control. The tumor dimensions and body weights were measured every other day. The tumor volume was calculated according to a standard formula: *volume=*width^2^ × length/2. The experiment was terminated and the mice were killed when the tumor volumes in the solvent-treated group exceeded 1500 mm^3^. The mouse organs and blood were collected and used for hematoxylin–eosin staining and serum chemistry assay. TGI rates were assessed using the following formula: *TGI=1− (T*_*t*_*−T*_*0*_*)/(C*_*t*_*−C*_*0*_*) × 100%*. *T*_*t*_ and *T*_*0*_ indicate the tumor volumes of the treated group at days t and 0. *C*_*t*_ and *C*_*0*_ indicate the tumor volumes of the control group at days t and 0, respectively.

### P-gp ATPase activity assay

The P-gp ATPase activity assay was conducted using the P-gp Glo Assay System following the manufacturer's protocol (Promega Biotech, Madison, WI, USA). Na_3_VO_4_ (a P-gp inhibitor without P-gp ATPase stimulation activity), verapamil (a P-gp substrate with ATPase stimulation activity) and DMSO (the solvent for TBPT) were used as controls. TBPT (2 *μ*M) and the control reagents were incubated with the P-gp membrane for 1 h. Then, the luminescence was measured using a POLARstar Galaxy luminometer (BMG Labtech, Offenburg, Germany). The change in luminescence was calculated by comparing the Na_3_VO_4_-treated samples with the other compound-treated samples. The change in luminescence change is proportional to the ATP consumption in the samples, which correlated positively with the P-gp ATPase activity.

### Genome-wide transcriptional profiling microarray

Cells (1 × 10^6^ cells) were seeded and incubated in 10 cm culture plates for 24 h. After the cells were treated with DMSO or TBPT (2 *μ*M) for 4 h, they were washed in PBS and suspended in 1.0 ml TRIzol reagent (Life Technologies, Grand Island, NY, USA). RNA preparation and microarray hybridization were performed according to the manufacturer's instructions (Kangchen Bio-Tech, Shanghai, China) for the Agilent Whole Human Genome Oligo Microarray Platform (Santa Clara, CA, USA).^20^

### Tubulin polymerization assay

A fluorescence-based tubulin polymerization assay kit was used to monitor the polymerization process according to the manufacturer's instructions (Cytoskeleton, Denver, CO, USA). Briefly, after pre-warming the assay plate at 37 °C for 1 min, DMSO (control, 0.1%), colchicine (5 *μ*M) or TBPT (0.1–100 *μ*M) was added to each well. Tubulin solution (50 *μ*l) was dispensed rapidly into each well. Then, the fluorescence of samples in the assay plates was measured every 2 min for 50 min at 37 °C using a 1420 Multi-label Counter (Perkin Elmer, Boston, MA, USA). The excitation wavelength was 355 nm, and the emission wavelength was 460 nm.

### Immunofluorescence staining for microtubule structure

Cells (1 × 10^5^/ml) were seeded in 24-well culture plates and treated with DMSO, TBPT (2 *μ*M) or colchicine (1 *μ*M) on the next day for 24 h. The cells were washed in PBS and then fixed in paraformaldehyde (4% v/v) for 10 min. After the cells were washed three times in PBS, they were blocked with 5% (w/v) BSA for 30 min at RT. Then, *α*-tubulin antibody (CST, Beverly, MA, USA) was incubated with cells in a humid chamber overnight at 4 °C. After the cells were washed three times in PBS, they were incubated with goat anti-rabbit IgG-FITC (Jackson ImmunoResearch, West Grove, PA, USA) for 1 h at 37 °C. Then, the cells were mounted with mounting medium containing DAPI (Vector Lab, Peterborough, UK) after they were washed three times in PBS. Finally, the cells were observed and imaged using a fluorescence microscope (Olympus IX71, Tokyo, Japan).

### Molecular docking study

Molecular operating environment (MOE) software package (Chemical Computing Group Inc., Montréal, Quebec, Canada) was used to perform the docking study. The 2D SMILES of compounds were converted to energy-minimized 3D structures, and partial charges were calculated using MOE's Rebuild 3D procedure with a root-mean-square gradient of 0.1 in a Merck Molecular Force Field 94x. The 3D tubulin crystal structure of each compound was downloaded from the Protein Data Bank (ID: 1SA0). The structure was loaded into MOE, and implicit hydrogens were added and ionization states were assigned using MOE's Protonate 3D procedure. The active site was defined by the formation of the co-crystalized ligand colchicine on tubulin. Compounds were docked to the active site using MOE's triangle matcher algorithm, and the poses were ranked in terms of their ASE scoring. Poses were relaxed by forcefield refinement in an AMBER94 forcefield and ranked by affinity dG score (S score). MOE's affinity dG scoring function estimates the contribution to free energy of binding from ionic, hydrophobic, or hydrogen bonds between ligand and receptor atoms. To correlate the S score to an experimentally determined conformation, the conformation closest to the crystalized structure according to the lowest root-mean-square deviation of atomic positions was chosen. The conformations for compounds were ranked in terms of their S scores and the conformation with the lowest S score was selected.

### Flow cytometric analysis

Cell cycle and apoptosis analyses were performed on a Guava EasyCyte Flow Cytometry System (Millipore, Billerica, MA, USA). Cells were seeded in 6-cm culture plates and treated with 2 *μ*M TBPT or an equal volume of DMSO on the next day for the indicated times. To analyze the intracellular DNA content, cells were harvested, washed twice in PBS and fixed in 70% ethanol at −20 °C overnight. After the cells were washed in PBS, they were stained with Guava cell cycle reagent (Millipore) and analyzed via flow cytometry. For cell apoptosis analysis, the cells were harvested, washed in PBS, stained with PI/Annexin V-FITC reagent (Life Technologies) and analyzed by flow cytometry.

### Western blot analysis

Cells (1 × 10^6^ cells in 10 ml medium) were seeded in 10-cm dishes. After the NSCLC cells were treated with DMSO or TBPT, they were washed and collected. Cell lysates were prepared conventionally in RIPA lysis buffer. The protein concentrations were determined using the BCA protein assay. Equal amounts of total protein were loaded onto SDS-PAGE gels (8–10%) and transferred onto PVDF membranes. After the membranes were blocked with 5% nonfat milk for 1 h, they were blotted with primary antibodies against P-gp, *β*-actin, full-length caspase 3 (Santa Cruz Biotechnology, Dallas, TX, USA), caspase 8 (BD Biosciences, San Jose, CA, USA), p21, caspase 9, cleaved caspase 3 and PARP (CST) at 1 : 500–1 : 1000 dilution overnight at 4 °C. Then, the membranes were incubated with goat anti-mouse or goat anti-rabbit secondary antibodies (1 : 5000, Santa Cruz) at RT for 1 h. An enhanced chemiluminescence western blot system (Millipore) was used to detect the immunoreactive bands. Intensity of the blot was determined using the ImageJ software (NIH, Bethesda, MD, USA).

### Caspases activity assay

The activities of caspases 8, 9, and 3/7 in H460_TaxR_ and H460 cells were examined via a fluorescence-based assay using Caspase-Glo 8, Caspase-Glo 9 Assay kits and Apo-ONE Homogeneous Caspase-3/7 Assay Kit (Promega, Madison, WI, USA). Cells (1 × 10^6^ cells in 10 ml medium) were seeded in 10-cm dishes. After 24 h, the cells were treated with TBPT (2 *μ*M) or DMSO for 6, 12 and 24 h. Then, the cells were washed in PBS and collected. Cell lysates were prepared conventionally in RIPA lysis buffer. Protein concentrations were determined using the BCA protein assay. Equal amounts (10 *μ*g) of total protein of samples were added to 96-well culture plates containing 100 *μ*l caspases 8, 9 and 3/7 activity working solution. After the samples were incubated for 1 h at RT, the fluorescence of each well was determined at 499 nm with an emission wavelength of 521 nm using a multimode plate reader (Victor X2, Perkin Elmer, Boston, MA, USA). The fluorescence intensities are proportional to the activities of caspases 8, 9 and 3/7.

### Mitochondrial membrane potential determination assay

The mitochondrial membrane potential was determined using JC-1 dye (Beyotime, Beijing, China). NSCLC cells (4 × 10^4^ cells in 0.5 ml) were seeded in 24-well culture plates. The cells were treated with DMSO or TBPT (2 *μ*M) on the next day for 12 h. Carbonyl cyanide m-chlorophenylhydrazone (CCCP; 10 *μ*M), a mitochondrial membrane disrupter, was used to treat cells for 20 min as a positive control. Then, the cells were incubated with 200 *μ*l JC-1 working solution at 37 °C for 20 min. After the cells were washed in RPMI-1640 medium, they were observed and imaged using a fluorescence microscope (Olympus IX71).

### Kinome screening assay

The binding assay for 442 non-mutant kinases was performed using KINOMEscan (DiscoveRx Corporation, Fremont, CA, USA) as reported previously.^58, 59^ TBPT was screened at concentrations of 5 and 10 *μ*M to identify the affected kinases. KINOMEscan is based on a competition reaction that quantitatively measures the ability of a test compound to compete with an active site-directed ligand for binding to the kinase active site. Binding reactions were performed using DNA-tagged kinases, immobilized affinity ligand and TBPT in binding buffer (20% SeaBlock, 0.17 × PBS, 0.05% Tween 20 and 6 mM DTT) at RT. After the assay plates were incubated with shaking for 1 h, the liganded beads were washed with washing buffer (1 × PBS and 0.05% Tween 20). Then, the beads were re-suspended in elution buffer (1 × PBS, 0.05% Tween 20, and 0.5 *μ*M ligand) and incubated with shaking for 30 min. The kinase concentration in the eluates was measured via qPCR. The results are represented as ‘%Ctrl', which was calculated using the following formula: (test compound signal–positive control signal)/(DMSO signal–positive control signal) × 100%. A lower % Ctrl indicates stronger binding to the kinase active site.

### Statistical analysis

Data are expressed as the mean±S.D. The statistical significance was tested using an unpaired two-tailed Student's *t*-test. *P*-values < 0.05 were considered to indicate statistically significant differences.

## Figures and Tables

**Table 1:** Drug-like properties predicted by *in vitro* assays^a^

**ADME assays**	**TBPT**	**Criterion**
PAMPA Pe (10^−6^cm/s)	1081.1±30.2	>200
Caco-2 P_app_ A/B (nm/s)	344.79±20.6	NA
Caco-2 P_app_ B/A (nm/s)	206.23±70.05	NA
Efflux ratio (B2A/A2B)	0.59±0.17	<2
T_1/2_ in human liver microsome	5.18±0.69 h	0.1–24 h
T_1/2_ in human plasma	>50 h	>24 h
Plasma protein binding	98.36±0.17%	<99%

Abbreviation: NA, not applicable

a The results represent the mean±S.D., *N*≥2

## Abbreviations

P-gp: P-glycoprotein
PTX: paclitaxel
NSCLC: non-small-cell lung cancer
NHFB: normal human fibroblast
DMSO: dimethyl sulfoxide
EC_50_: half maximal effective concentration
IC_50_: half maximal inhibitory concentration
CCCP: carbonyl cyanide m-chlorophenylhydrazone
TGI: tumor growth inhibition
