# Supplementary material for: From fighting depression to conquering tumors: a novel tricyclic thiazepine compound as a tubulin polymerization inhibitor
Source: Cell Death Dis. 2016 Mar 17;7(3):e2143–. doi: 10.1038/cddis.2016.53 (PMC4823954; doi:10.1038/cddis.2016.53)
Supplement: Supplementary Information [file cddis201653x1.doc]

Supporting Information for:

**From fighting depression to conquering tumors: a novel tricyclic thiazepine compound as a tubulin polymerization inhibitor**

Yan Mu, Yongjin Liu, Jinbao Xiang, Qiu Zhang, Shumei Zhai, Daniel P. Russo, Hao Zhu and Xu Bai and Bing Yan*

**Contents**

**Figure S1. The relative organ weight of mice.**

**Figure S2. The effects of TBPT on tubulin polymerization compared with PTX and Colchicine.**

**Figure S3. The cell cycle and apoptosis analysis of NHFB cells.**

**Figure S4. The cell cycle and apoptosis analysis of mice hippocampus cells.**

**Table S1. Cytotoxicity of several clinical drugs and TBPT.**

**Table S2. The differentially expressed genes involved in crucial biological process in H460_TaxR_ cells after TBPT-treatment.**

**Table S3. The inhibitory bindings of TBPT to 442 kinases in human kinome.**

**
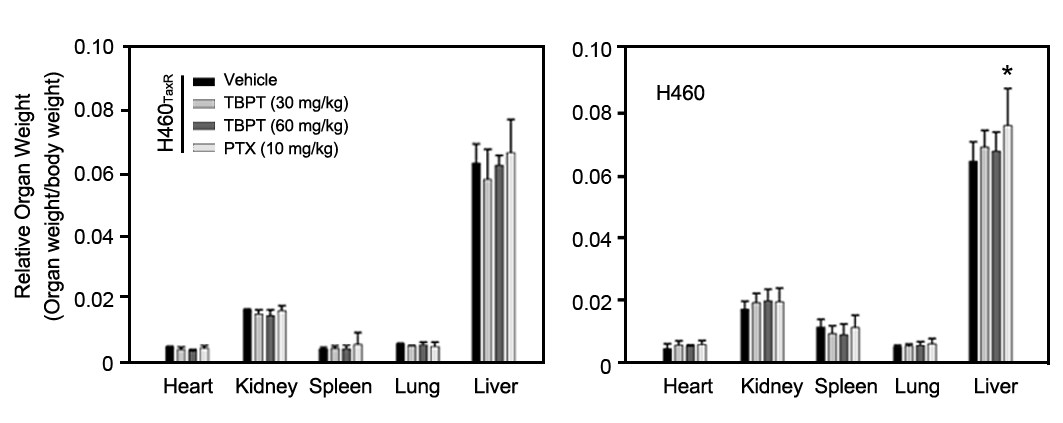
**

**Figure S1.** The relative organ weight of mice treated with TBPT or paclitaxel at the end of experiments. The weights of major organs were measured after desiccation with bibulous paper. *, *P*<0.05, Student’s *t-*test with vehicle group, n = 5.

**
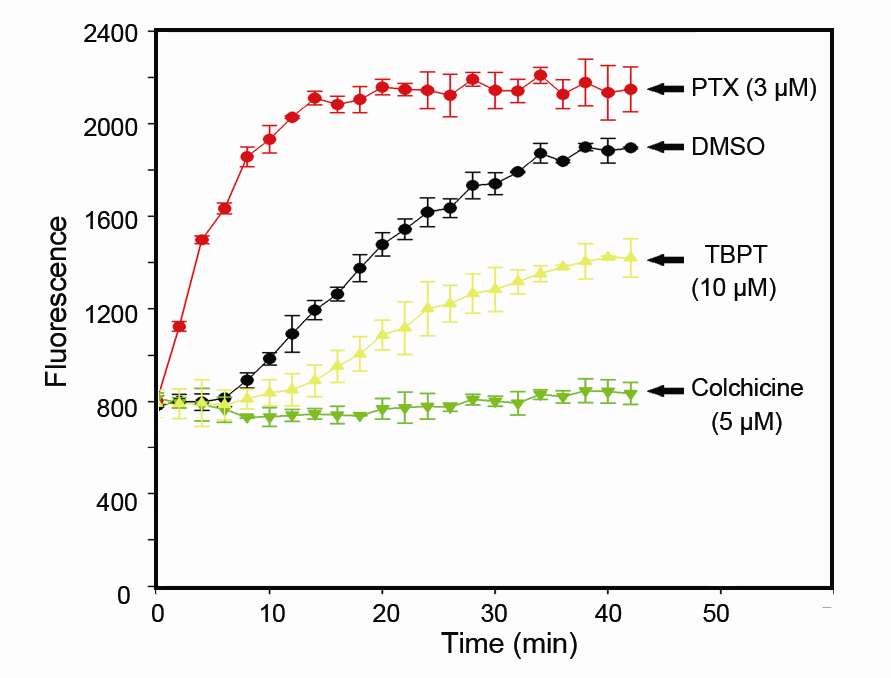
**

**Figure S2.** The effects of TBPT on tubulin polymerization compared with PTX and Colchicine.


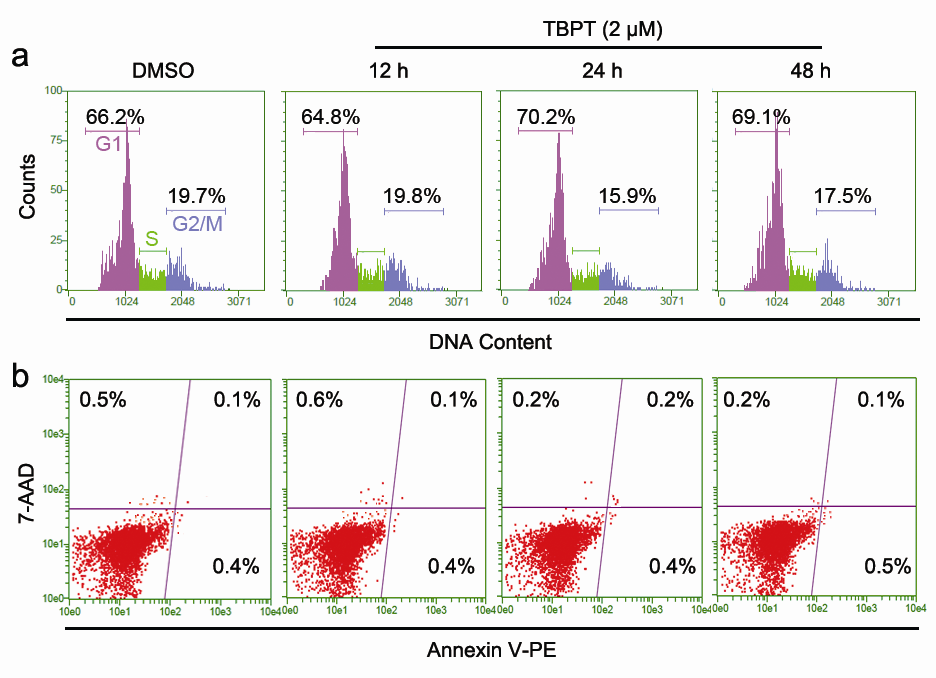


**Figure S3.** The cell cycle (a) and apoptosis (b) analysis of NHFB cells. Cells were treated with DMSO or TBPT (2 μM) for the indicated times. After the cells were stained, they were analyzed for cell cycle and apoptosis using flow cytometry.


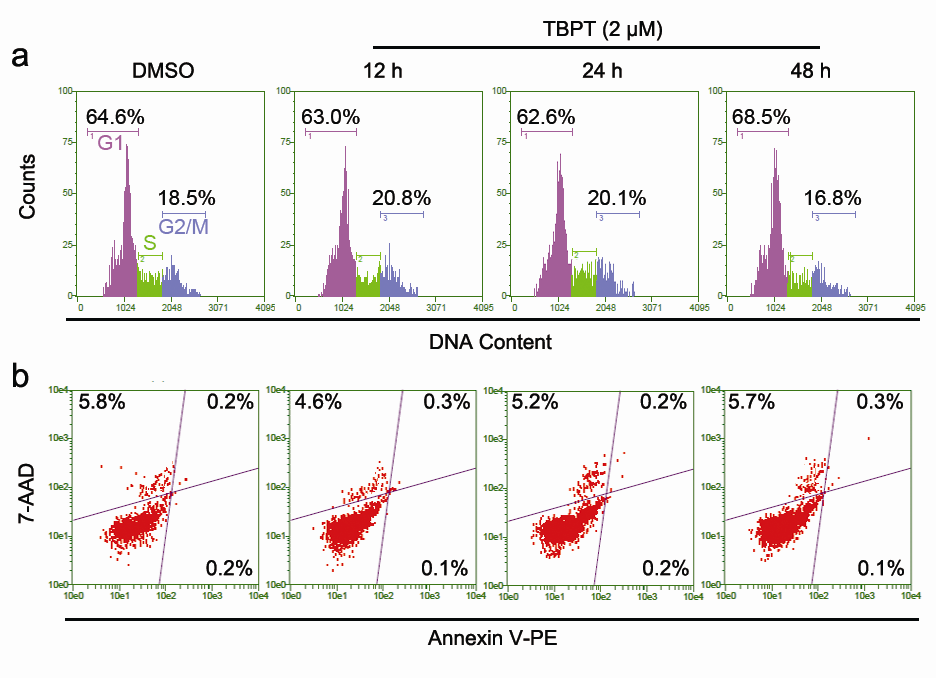


**Figure S4.** The cell cycle (a) and apoptosis (b) analysis of mice hippocampus cells. The new-born mice hippocampus cells were derived and cultured as previously report (F Lu et al. *Cell Death and Disease*, 2012). Cells were treated with DMSO or TBPT (2 μM) for the indicated times. After the cells were stained, they were analyzed for cell cycle and apoptosis using flow cytometry.

**Table S1. Cytotoxicity of several clinical drugs and TBPT*^a^***

| Agents | EC_50_ | | | Resistance  Folds*^a^* |
| --- | --- | --- | --- | --- |
|  | H460 | H460_TaxR_ | H460_TaxR_(+Rev) |  |
| VCR (nM) | 12.9±5.2 | 1011.6±100.8 | 9.1±3.3 | 78.4 |
| PTX (nM) | 6.0±2.1 | 990.0±190.4 | 4.4±2.8 | 165.0 |
| DOX (μM) | 0.11±0.07 | 2.05±0.69 | 0.17±0.10 | 18.6 |
| TBPT (μM) | 0.45±0.19 | 0.60±0.31 | 0.61±0.26 | 1.3 |

*^a^*The results represent the mean ± SD, N ≥ 2. The resistance folds are calculated by dividing the EC_50_ in H460_TaxR_ cells by the EC_50_ in H460 cells. VCR, Vincristine; PTX, Paclitaxel; DOX, Doxorubicin; Rev, Reversan (P-gp inhibitor).

**Table S2. The differentially expressed genes involved in crucial biological process in H460_TaxR_ cells after TBPT-treatment*^a^***

| Cell Component | Biological Process | Differentially Expressed Genes in H460_TaxR_ |
| --- | --- | --- |
| Microtubule  Cytoskeleton | Microtubule Cytoskeleton Organization | *DYNLT1 (-2.45), TTK (-4.71), MAP4 (-2.22), CNTLN(-2.67), ROCK2(-2.38), GCC2 (-2.75), DST(-2.31), PCM1(-2.12), RSPH9(-2.28), NEK7(-2.24), FMN2(-3.01), BRCA2 (-2.03)* |
|  | Microtubule-based Movement | *DYX1C1 (-2.13), RSPH9 (-2.28), DST (-2.31), PCM1 (-2.12), IFT74 (-3.10), MAP4 (-2.22), FMN2 (-3.01), ARHGAP21 (-2.16), KIF20B (-3.01)* |
|  | Establishment of Spindle Localization | *DYNLT1 (-2.45), MAP4 (-2.22), FMN2 (-3.01)* |
|  | Microtubule Organizing Center Organization | *CNTLN (-2.67), ROCK2 (-2.38), PCM1 (-2.12), BRCA2 (-2.03), GCC2 (-2.75)* |
|  | Mitotic Cell Cycle Process | *PSMA1 (-2.42), PSMC6 (-2.76), RPS6 (-2.36), CCNE2 (-3.36), AKAP9 (-2.44), CENPF (-2.30), EP300 (-2.47), PCM1 (-2.12), CEP70 (-2.54), DYNLT1 (-2.45), MAP4 (-2.22), TTK (-4.71), USP16 (-2.82), HGF (-3.17), NEK1 (-2.18), FIGN (-2.26), MIS18BP1 (-2.67), DYNLT3 (-3.96), NUF2 (-3.46), LRRCC1 (-2.71), KIF20B (-3.01), SEPT7 (-2.58), SMC2 (-2.01), ANXA1 (-2.83)* |
|  |  |  |
|  |  |  |
| Nucleus | Nuclear Division | *USP16 (-2.82), CENPF (-2.30), HGF (-3.17), NEK1(-2.18), FIGN (-2.26), MIS18BP1 (-2.67), RPS6 (-2.36), DYNLT3 (-3.96), DYNLT1 (-2.45), NUF2 (-3.46), LRRCC1 (-2.71), KIF20B (-3.01), SEPT7 (-2.58), SMC2 (-2.01), TTK (-4.71), MNS1(-3.37), BRCA2 (-2.03), DAZL (-3.07), FMN2 (-3.01), SGOL2 (-2.58)* |
|  | Chromosome Segregation | *ESCO1 (-3.01), SMC2 (-2.01), TTK (-4.71), CENPF (-2.30), FMN2 (-3.01), SGOL2 (-2.58), NUF2 (-3.46)* |
|  | DNA metabolic process | *LIG4(-2.26), CENPF (-2.30), DNAJC2 (-2.29), RECQL (-2.19),* *SMC6 (-2.97), BRCA2 (-2.03),* *TATDN1 (-2.86), ATRX (-2.56), MIS18BP1 (-2.67), CENPK (-2.92), HIST2H2BE (-2.14), CCNE2 (-3.36), ESCO1 (-3.01), USP1 (-2.66), SETD2 (-2.16), PMS1 (-2.50), HAT1 (-2.04), SMC2 (-2.01), NYNRIN (-2.68),* *CHD1 (-2.09),* *TET1 (-2.25), AICDA (-2.12), KITLG (-2.05), HGF (-3.17), NOX4 (-2.09),* *DEK (-2.97)* |
|  | Regulation of RNA Metabolic Process | *ZNF family (50 genes -2.02 ~ -4.11), EP300 (-2.47), HIVEP1 (-2.12), PPM1A (-2.09), COPS2 (-4.95), RBM11 (-2.15), HAT1 (-2.04), SOX21 (-2.63), SETD2 (-2.16), HOXB8 (-2.43), ZBTB41 (-2.85), NEO1 (-3.46), NFYB (-2.15), ESF1 (-2.31), ATRX (-2.56), BDP1 (-2.46), PRKCQ (-3.00), SF1 (-2.03), HMGN5 (-4.51), CHD9 (-2.09), CLOCK (-2.19), RB1CC1 (-2.48), USP16 (-2.83), SUB1 (-2.42), CHD1 (-2.09), DBX1 (-2.45), LPIN1 (-2.17), BRWD1 (-2.86), DEK (-2.97), BLZF1 (-2.41), CIR1 (-2.37), POLR3G (-2.13), DNAJC2 (-2.29), ROR2 (-2.28), BRCA2 (-2.03), CENPF (-2.30), DEPDC1 (-2.20), ARID4A (-2.15), NFE2L2 (-2.29), CLK4 (-2.52), CDX1 (-2.17), HGF (-3.17), HOXD10 (-3.03), NHLH2 (-2.09), CENPK (-2.92), IFT74 (-3.10), TET1 (-2.25), CWC22 (-2.53), LTF (-4.63), AICDA (-2.12)* |
|  | Chromatin Organization | *ATRX (-2.56), MIS18BP1 (-2.67), CENPK (-2.92), HIST2H2BE (-2.14), SETD2 (-2.16), HAT1 (-2.04), CHD1 (-2.09), DNAJC2 (-2.29), DEK (-2.97), HMGN5 (-4.51), CHD9 (-2.09), TET1 (-2.25), CLOCK (-2.19), USP16 (-2.83), ARID4A (-2.15), BRCA2 (-2.03), EP300 (-2.47), AICDA (-2.12)* |
| Others Down- regulated | Negative Regulation of Apoptotic Process | *KITLG (-2.05), LIG4 (-2.26), ROCK1 (-2.23), PRKCQ (-3.00), CCL19 (-2.08), API5 (-2.39), NFE2L2 (-2.29), SCG2 (-2.12), ZNF268 (-2.17), PDCD10 (-2.05), ANXA1 (-2.83), HGF (-3.17), IL6ST (-2.00), LTF (-4.63), FMN2 (-3.01), PSMA1 (-2.42), PSMC6 (-2.76), RPS6 (-2.36), BIRC7 (-3.37), ATG5 (-2.05), RB1CC1 (-2.48)* |
|  | NFκB Import into Nucleus | *ZNF268 (-2.17), CCL19 (-2.08), PPM1A (-2.09)* |
|  |  |  |
|  |  |  |
|  | Organelle Localization | *NMD3 (-2.71), DYNLT1 (-2.45), EEA1 (-2.07), PCM1 (-2.12), SHROOM2 (-2.51), TMEM106B (-2.54), MAP4 (-2.22), FMN2 (-3.01), CENPF (-2.30), ARHGAP21 (-2.16)* |
| Ion Channel Complex | Ion Transport | *SHANK2 (2.48), GABRB1 (2.19), GABRP (2.10), CLCA3P (2.22), SCN3A (2.10), SCN8A (3.21), KCNG2 (2.60), CATSPER1 (3.17), ATP12A (2.49)* |
| Others Up-regulated | Neurological System Process | *GNAT1 (2.92), NPAS3 (2.66), GSX2 (4.99), KERA (2.37), CRYAA (2.24), CLDN19 (2.53), PDE6G (2.02), CDH23 (2.25), COL11A2 (2.15), LRTOMT (2.79), SLC26A5 (2.63), ALDH7A1 (2.00), SCN8A (3.21), EGR2 (2.13), SHANK2 (2.48), GRM5 (2.06), PRX (2.09), SCN3A (2.10), P2RX7 (2.68), ERBB2 (2.10), STRC (2.49), HPN (2.02), Olfactory Receptor family (7 genes, 2.07~3.11 )* |
|  | Negative Regulation of Mononuclear Cell Proliferation | *ERBB2 (2.10), IL2RA (2.34), PDE5A (3.14)* |
|  | Regulation of Phosphatase Activity | *HPN (2.02), PCDH11X (2.58), SLC7A14 (2.03), PLEK (2.49)* |
|  | Positive Regulation of Protein Phosphorylation | *GRM5 (2.06), GHR (5.97), PDE6G (2.02), MADD (2.15), EREG (2.41), NRG3 (2.16), GDF7 (2.16), IL24 (2.09), ERBB2 (2.10), PDE5A (3.14), CCL21 (2.82), PROM2 (2.43), WNT1 (2.01)* |
|  | BMP Signaling Pathway | *WNT1 (2.01), CHRDL1 (2.43), GDF7 (2.16), USP9Y (2.69)* |

*^a^*The blue were downregulated issues. The red were upregulated issues. The number in the bracket is the fold change of the gene expression.

**Table S3 The inhibitory bindings of TBPT to 442 kinases in human kinome*^a^***

| Kinases screened | Percent Control (% Ctrl)  @ 5 μM* | Percent Control (% Ctrl)  @ 10 μM* |
| --- | --- | --- |
| AAK1 | 100 | 89 |
| ABL1(E255K)-phosphorylated | 60 | 89 |
| ABL1(F317I)-nonphosphorylated | 89 | 100 |
| ABL1(F317I)-phosphorylated | 78 | 81 |
| ABL1(F317L)-nonphosphorylated | 80 | 100 |
| ABL1(F317L)-phosphorylated | 72 | 98 |
| ABL1(H396P)-nonphosphorylated | 62 | 77 |
| ABL1(H396P)-phosphorylated | 68 | 92 |
| ABL1(M351T)-phosphorylated | 81 | 100 |
| ABL1(Q252H)-nonphosphorylated | 79 | 100 |
| ABL1(Q252H)-phosphorylated | 83 | 100 |
| ABL1(T315I)-nonphosphorylated | 100 | 100 |
| ABL1(T315I)-phosphorylated | 100 | 100 |
| ABL1(Y253F)-phosphorylated | 70 | 95 |
| ABL1-nonphosphorylated | 89 | 100 |
| ABL1-phosphorylated | 72 | 97 |
| ABL2 | 80 | 97 |
| ACVR1 | 100 | 100 |
| ACVR1B | 98 | 91 |
| ACVR2A | 100 | 93 |
| ACVR2B | 87 | 82 |
| ACVRL1 | 100 | 71 |
| ADCK3 | 98 | 100 |
| ADCK4 | 83 | 94 |
| AKT1 | 84 | 100 |
| AKT2 | 76 | 100 |
| AKT3 | 100 | 100 |
| ALK | 100 | 71 |
| AMPK-alpha1 | 100 | 100 |
| AMPK-alpha2 | 81 | 95 |
| ANKK1 | 91 | 72 |
| ARK5 | 100 | 100 |
| ASK1 | 100 | 100 |
| ASK2 | 82 | 100 |
| AURKA | 100 | 100 |
| AURKB | 98 | 92 |
| AURKC | 85 | 96 |
| AXL | 98 | 100 |
| BIKE | 87 | 93 |
| BLK | 88 | 88 |
| BMPR1A | 97 | 80 |
| BMPR1B | 95 | 88 |
| BMPR2 | 85 | 100 |
| BMX | 92 | 93 |
| BRAF | 100 | 100 |
| BRAF(V600E) | 94 | 94 |
| BRK | 100 | 98 |
| BRSK1 | 100 | 97 |
| BRSK2 | 100 | 100 |
| BTK | 91 | 83 |
| CAMK1 | 96 | 98 |
| CAMK1D | 81 | 91 |
| CAMK1G | 100 | 100 |
| CAMK2A | 100 | 87 |
| CAMK2B | 100 | 100 |
| CAMK2D | 94 | 100 |
| CAMK2G | 100 | 96 |
| CAMK4 | 100 | 93 |
| CAMKK1 | 100 | 92 |
| CAMKK2 | 97 | 83 |
| CASK | 100 | 100 |
| CDC2L1 | 99 | 100 |
| CDC2L2 | 100 | 100 |
| CDC2L5 | 100 | 97 |
| CDK11 | 100 | 100 |
| CDK2 | 98 | 93 |
| CDK3 | 100 | 100 |
| CDK4-cyclinD1 | 100 | 79 |
| CDK4-cyclinD3 | 100 | 90 |
| CDK5 | 85 | 98 |
| CDK7 | 83 | 100 |
| CDK8 | 94 | 100 |
| CDK9 | 100 | 100 |
| CDKL1 | 94 | 88 |
| CDKL2 | 95 | 100 |
| CDKL3 | 77 | 74 |
| CDKL5 | 100 | 100 |
| CHEK1 | 100 | 100 |
| CHEK2 | 100 | 100 |
| CIT | 98 | 97 |
| CLK1 | 95 | 100 |
| CLK2 | 100 | 100 |
| CLK3 | 100 | 100 |
| CLK4 | 93 | 96 |
| CSF1R | 93 | 100 |
| CSK | 96 | 100 |
| CSNK1A1 | 100 | 86 |
| CSNK1A1L | 100 | 91 |
| CSNK1D | 98 | 90 |
| CSNK1E | 100 | 96 |
| CSNK1G1 | 100 | 89 |
| CSNK1G2 | 100 | 100 |
| CSNK1G3 | 100 | 98 |
| CSNK2A1 | 100 | 84 |
| CSNK2A2 | 100 | 100 |
| CTK | 94 | 100 |
| DAPK1 | 83 | 100 |
| DAPK2 | 100 | 99 |
| DAPK3 | 100 | 100 |
| DCAMKL1 | 100 | 89 |
| DCAMKL2 | 100 | 100 |
| DCAMKL3 | 100 | 79 |
| DDR1 | 100 | 92 |
| DDR2 | 100 | 99 |
| DLK | 88 | 81 |
| DMPK | 88 | 100 |
| DMPK2 | 100 | 83 |
| DRAK1 | 94 | 92 |
| DRAK2 | 100 | 91 |
| DYRK1A | 88 | 100 |
| DYRK1B | 99 | 100 |
| DYRK2 | 93 | 100 |
| EGFR | 100 | 91 |
| EGFR(E746-A750del) | 81 | 100 |
| EGFR(G719C) | 73 | 100 |
| EGFR(G719S) | 83 | 94 |
| EGFR(L747-E749del, A750P) | 91 | 91 |
| EGFR(L747-S752del, P753S) | 76 | 91 |
| EGFR(L747-T751del,Sins) | 88 | 98 |
| EGFR(L858R) | 85 | 100 |
| EGFR(L858R,T790M) | 78 | 88 |
| EGFR(L861Q) | 100 | 100 |
| EGFR(S752-I759del) | 79 | 100 |
| EGFR(T790M) | 100 | 96 |
| EIF2AK1 | 100 | 100 |
| EPHA1 | 100 | 94 |
| EPHA2 | 87 | 95 |
| EPHA3 | 80 | 100 |
| EPHA4 | 100 | 96 |
| EPHA5 | 92 | 100 |
| EPHA6 | 98 | 92 |
| EPHA7 | 93 | 100 |
| EPHA8 | 96 | 93 |
| EPHB1 | 100 | 100 |
| EPHB2 | 91 | 100 |
| EPHB3 | 96 | 100 |
| EPHB4 | 100 | 98 |
| EPHB6 | 99 | 100 |
| ERBB2 | 100 | 100 |
| ERBB3 | 45 | 100 |
| ERBB4 | 93 | 99 |
| ERK1 | 100 | 100 |
| ERK2 | 76 | 92 |
| ERK3 | 100 | 100 |
| ERK4 | 89 | 100 |
| ERK5 | 100 | 96 |
| ERK8 | 94 | 100 |
| ERN1 | 92 | 100 |
| FAK | 92 | 92 |
| FER | 93 | 100 |
| FES | 100 | 98 |
| FGFR1 | 98 | 100 |
| FGFR2 | 93 | 100 |
| FGFR3 | 89 | 100 |
| FGFR3(G697C) | 100 | 82 |
| FGFR4 | 96 | 97 |
| FGR | 97 | 73 |
| FLT1 | 100 | 98 |
| FLT3 | 100 | 68 |
| FLT3(D835H) | 100 | 97 |
| FLT3(D835Y) | 92 | 100 |
| FLT3(ITD) | 95 | 100 |
| FLT3(K663Q) | 84 | 94 |
| FLT3(N841I) | 85 | 100 |
| FLT3(R834Q) | 89 | 100 |
| FLT4 | 100 | 100 |
| FRK | 100 | 99 |
| FYN | 85 | 95 |
| GAK | 100 | 90 |
| GCN2(Kin.Dom.2,S808G) | 100 | 100 |
| GRK1 | 100 | 100 |
| GRK4 | 100 | 95 |
| GRK7 | 100 | 100 |
| GSK3A | 100 | 100 |
| GSK3B | 89 | 100 |
| HCK | 93 | 100 |
| HIPK1 | 71 | 63 |
| HIPK2 | 100 | 100 |
| HIPK3 | 100 | 100 |
| HIPK4 | 91 | 100 |
| HPK1 | 93 | 100 |
| HUNK | 89 | 84 |
| ICK | 100 | 100 |
| IGF1R | 100 | 79 |
| IKK-alpha | 56 | 75 |
| IKK-beta | 84 | 98 |
| IKK-epsilon | 80 | 100 |
| INSR | 98 | 84 |
| INSRR | 100 | 100 |
| IRAK1 | 100 | 93 |
| IRAK3 | 92 | 82 |
| IRAK4 | 100 | 88 |
| ITK | 100 | 90 |
| JAK1(JH1domain-catalytic) | 95 | 89 |
| JAK1(JH2domain-pseudokinase) | 100 | 100 |
| JAK2(JH1domain-catalytic) | 84 | 98 |
| JAK3(JH1domain-catalytic) | 100 | 100 |
| JNK1 | 100 | 85 |
| JNK2 | 99 | 100 |
| JNK3 | 84 | 95 |
| KIT | 72 | 100 |
| KIT(A829P) | 95 | 96 |
| KIT(D816H) | 71 | 100 |
| KIT(D816V) | 62 | 99 |
| KIT(L576P) | 78 | 100 |
| KIT(V559D) | 72 | 91 |
| KIT(V559D,T670I) | 94 | 100 |
| KIT(V559D,V654A) | 97 | 90 |
| LATS1 | 100 | 100 |
| LATS2 | 100 | 100 |
| LCK | 97 | 100 |
| LIMK1 | 100 | 100 |
| LIMK2 | 91 | 100 |
| LKB1 | 100 | 89 |
| LOK | 100 | 92 |
| LRRK2 | 94 | 100 |
| LRRK2(G2019S) | 86 | 99 |
| LTK | 100 | 100 |
| LYN | 87 | 100 |
| LZK | 100 | 93 |
| MAK | 94 | 100 |
| MAP3K1 | 100 | 100 |
| MAP3K15 | 100 | 100 |
| MAP3K2 | 82 | 100 |
| MAP3K3 | 79 | 92 |
| MAP3K4 | 99 | 96 |
| MAP4K2 | 92 | 100 |
| MAP4K3 | 75 | 91 |
| MAP4K4 | 94 | 63 |
| MAP4K5 | 100 | 85 |
| MAPKAPK2 | 100 | 100 |
| MAPKAPK5 | 100 | 88 |
| MARK1 | 89 | 100 |
| MARK2 | 86 | 84 |
| MARK3 | 96 | 87 |
| MARK4 | 81 | 100 |
| MAST1 | 45 | 87 |
| MEK1 | 100 | 92 |
| MEK2 | 92 | 100 |
| MEK3 | 100 | 100 |
| MEK4 | 82 | 100 |
| MEK5 | 100 | 95 |
| MEK6 | 83 | 100 |
| MELK | 100 | 100 |
| MERTK | 100 | 80 |
| MET | 100 | 92 |
| MET(M1250T) | 100 | 77 |
| MET(Y1235D) | 89 | 85 |
| MINK | 89 | 83 |
| MKK7 | 100 | 100 |
| MKNK1 | 36 | 100 |
| MKNK2 | 100 | 96 |
| MLCK | 92 | 95 |
| MLK1 | 100 | 100 |
| MLK2 | 70 | 100 |
| MLK3 | 87 | 100 |
| MRCKA | 89 | 99 |
| MRCKB | 89 | 92 |
| MST1 | 93 | 100 |
| MST1R | 80 | 100 |
| MST2 | 63 | 100 |
| MST3 | 95 | 96 |
| MST4 | 63 | 99 |
| MTOR | 100 | 95 |
| MUSK | 100 | 100 |
| MYLK | 100 | 81 |
| MYLK2 | 100 | 82 |
| MYLK4 | 94 | 99 |
| MYO3A | 100 | 90 |
| MYO3B | 100 | 100 |
| NDR1 | 100 | 87 |
| NDR2 | 100 | 100 |
| NEK1 | 90 | 100 |
| NEK11 | 43 | 100 |
| NEK2 | 92 | 81 |
| NEK3 | 100 | 100 |
| NEK4 | 65 | 99 |
| NEK5 | 100 | 100 |
| NEK6 | 96 | 95 |
| NEK7 | 100 | 89 |
| NEK9 | 100 | 88 |
| NIM1 | 90 | 78 |
| NLK | 83 | 89 |
| OSR1 | 100 | 87 |
| p38-alpha | 63 | 100 |
| p38-beta | 100 | 76 |
| p38-delta | 75 | 65 |
| p38-gamma | 100 | 88 |
| PAK1 | 81 | 92 |
| PAK2 | 100 | 100 |
| PAK3 | 100 | 100 |
| PAK4 | 100 | 100 |
| PAK6 | 100 | 100 |
| PAK7 | 78 | 95 |
| PCTK1 | 90 | 100 |
| PCTK2 | 100 | 89 |
| PCTK3 | 82 | 100 |
| PDGFRA | 100 | 100 |
| PDGFRB | 68 | 100 |
| PDPK1 | 100 | 100 |
| PFCDPK1(P.falciparum) | 100 | 100 |
| PFPK5(P.falciparum) | 97 | 97 |
| PFTAIRE2 | 100 | 89 |
| PFTK1 | 100 | 100 |
| PHKG1 | 100 | 94 |
| PHKG2 | 100 | 100 |
| PIK3C2B | 100 | 100 |
| PIK3C2G | 100 | 100 |
| PIK3CA | 100 | 82 |
| PIK3CA(C420R) | 100 | 100 |
| PIK3CA(E542K) | 93 | 97 |
| PIK3CA(E545A) | 100 | 100 |
| PIK3CA(E545K) | 81 | 100 |
| PIK3CA(H1047L) | 100 | 100 |
| PIK3CA(H1047Y) | 95 | 100 |
| PIK3CA(I800L) | 98 | 72 |
| PIK3CA(M1043I) | 100 | 100 |
| PIK3CA(Q546K) | 100 | 94 |
| PIK3CB | 36 | 100 |
| PIK3CD | 94 | 100 |
| PIK3CG | 89 | 80 |
| PIK4CB | 100 | 93 |
| PIM1 | 91 | 90 |
| PIM2 | 100 | 100 |
| PIM3 | 96 | 100 |
| PIP5K1A | 98 | 100 |
| PIP5K1C | 100 | 94 |
| PIP5K2B | 100 | 100 |
| PIP5K2C | 100 | 100 |
| PKAC-alpha | 93 | 100 |
| PKAC-beta | 96 | 100 |
| PKMYT1 | 86 | 99 |
| PKN1 | 90 | 100 |
| PKN2 | 100 | 95 |
| PKNB(M.tuberculosis) | 100 | 85 |
| PLK1 | 76 | 80 |
| PLK2 | 100 | 83 |
| PLK3 | 95 | 75 |
| PLK4 | 100 | 92 |
| PRKCD | 72 | 100 |
| PRKCE | 100 | 100 |
| PRKCH | 82 | 100 |
| PRKCI | 95 | 91 |
| PRKCQ | 100 | 100 |
| PRKD1 | 100 | 78 |
| PRKD2 | 100 | 100 |
| PRKD3 | 100 | 78 |
| PRKG1 | 96 | 100 |
| PRKG2 | 88 | 98 |
| PRKR | 97 | 90 |
| PRKX | 92 | 70 |
| PRP4 | 99 | 100 |
| PYK2 | 100 | 100 |
| QSK | 76 | 100 |
| RAF1 | 100 | 91 |
| RET | 100 | 87 |
| RET(M918T) | 99 | 99 |
| RET(V804L) | 100 | 86 |
| RET(V804M) | 84 | 96 |
| RIOK1 | 83 | 76 |
| RIOK2 | 95 | 100 |
| RIOK3 | 100 | 100 |
| RIPK1 | 100 | 100 |
| RIPK2 | 92 | 97 |
| RIPK4 | 100 | 100 |
| RIPK5 | 58 | 83 |
| ROCK1 | 84 | 100 |
| ROCK2 | 100 | 92 |
| ROS1 | 100 | 100 |
| RPS6KA4(Kin.Dom.1-N-terminal) | 75 | 100 |
| RPS6KA4(Kin.Dom.2-C-terminal) | 100 | 90 |
| RPS6KA5(Kin.Dom.1-N-terminal) | 100 | 100 |
| RPS6KA5(Kin.Dom.2-C-terminal) | 100 | 93 |
| RSK1(Kin.Dom.1-N-terminal) | 100 | 74 |
| RSK1(Kin.Dom.2-C-terminal) | 100 | 98 |
| RSK2(Kin.Dom.1-N-terminal) | 98 | 100 |
| RSK3(Kin.Dom.1-N-terminal) | 100 | 82 |
| RSK3(Kin.Dom.2-C-terminal) | 100 | 100 |
| RSK4(Kin.Dom.1-N-terminal) | 83 | 94 |
| RSK4(Kin.Dom.2-C-terminal) | 94 | 57 |
| S6K1 | 100 | 88 |
| SBK1 | 100 | 85 |
| SgK110 | 100 | 100 |
| SGK3 | 100 | 99 |
| SIK | 92 | 92 |
| SIK2 | 84 | 90 |
| SLK | 100 | 86 |
| SNARK | 100 | 100 |
| SNRK | 100 | 100 |
| SRC | 87 | 100 |
| SRMS | 87 | 97 |
| SRPK1 | 88 | 67 |
| SRPK2 | 96 | 78 |
| SRPK3 | 100 | 89 |
| STK16 | 99 | 91 |
| STK33 | 99 | 100 |
| STK35 | 97 | 100 |
| STK36 | 84 | 100 |
| STK39 | 100 | 62 |
| SYK | 100 | 100 |
| TAK1 | 87 | 93 |
| TAOK1 | 100 | 100 |
| TAOK2 | 90 | 67 |
| TAOK3 | 89 | 100 |
| TBK1 | 100 | 100 |
| TEC | 90 | 99 |
| TESK1 | 87 | 95 |
| TGFBR1 | 100 | 85 |
| TGFBR2 | 100 | 81 |
| TIE1 | 100 | 98 |
| TIE2 | 100 | 91 |
| TLK1 | 93 | 97 |
| TLK2 | 100 | 100 |
| TNIK | 84 | 97 |
| TNK1 | 100 | 97 |
| TNK2 | 100 | 100 |
| TNNI3K | 100 | 89 |
| TRKA | 100 | 97 |
| TRKB | 96 | 100 |
| TRKC | 100 | 96 |
| TRPM6 | 100 | 85 |
| TSSK1B | 90 | 100 |
| TTK | 100 | 100 |
| TXK | 100 | 75 |
| TYK2(JH1domain-catalytic) | 69 | 100 |
| TYK2(JH2domain-pseudokinase) | 82 | 100 |
| TYRO3 | 99 | 100 |
| ULK1 | 100 | 83 |
| ULK2 | 91 | 92 |
| ULK3 | 100 | 100 |
| VEGFR2 | 95 | 84 |
| VRK2 | 100 | 69 |
| WEE1 | 100 | 96 |
| WEE2 | 100 | 100 |
| YANK1 | 96 | 100 |
| YANK2 | 99 | 100 |
| YANK3 | 88 | 96 |
| YES | 100 | 100 |
| YSK1 | 99 | 100 |
| YSK4 | 98 | 98 |
| ZAK | 94 | 83 |
| ZAP70 | 100 | 99 |

*^a^*Kinases with % Ctrl<35 are supposed to bind with the test compound at its active site, and kinases with % Ctrl<1 are supposed to bind with the test compound tightly.
